# Could Sarcopenia Be Related to Chemotherapy in Gastrointestinal Cancer? A Cross-Sectional Study Including Comprehensive Geriatric Assessment

**DOI:** 10.3390/jcm14030711

**Published:** 2025-01-22

**Authors:** Kamile Sılay, Gökhan Uçar, Tülay Eren, Hande Selvi Öztorun, Ozan Yazıcı, Nuriye Özdemir

**Affiliations:** 1 Department of Geriatrics, Faculty of Medicine, Ankara Bilkent City Hospital, Ankara Yıldırım Beyazıt University, 06800 Ankara, Türkiye; 2 Department of Medical Oncology, Ankara Bilkent City Hospital, 06800 Ankara, Türkiye; 3 Department of Medical Oncology, Ankara Etlik City Hospital, 06170 Ankara, Türkiye; 4 Department of Medical Oncology, Faculty of Medicine, Gazi University, 06810 Ankara, Türkiye

**Keywords:** sarcopenia, chemotherapy, gastrointestinal cancer, geriatric assessment

## Abstract

**Background:** Sarcopenia, which is characterized by the progressive loss of skeletal muscle mass, strength, and functionality, adversely affects cancer outcomes. This study aims to evaluate the development and progression of sarcopenia in patients with gastrointestinal cancer undergoing chemotherapy and its impact on comprehensive geriatric assessment outcomes in older participants. **Methods:** This cross-sectional study included 351 gastrointestinal cancer patients from October 2018 to December 2019. Pre- and post-chemotherapy measurements were taken for 243 participants. Sarcopenia was assessed using EWGSOP-2 criteria, including muscle mass, strength, and performance evaluations. A comprehensive geriatric assessment was conducted for patients aged 65 years and older. **Results:** The median age of participants was 57.84 years, with 31.7% being female and 29.2% being aged 65 years or older. A significant increase in the prevalence of sarcopenia post-chemotherapy was observed. The factors significantly associated with sarcopenia included low hand grip strength (−0.264; *p* < 0.001) and slow gait speed (0.222; *p* = 0.007). The muscle mass and albumin levels of older patients declined significantly post-treatment. **Conclusions:** This study highlights a strong association between chemotherapy and sarcopenia in gastrointestinal cancer patients, emphasizing the need for early detection and tailored interventions. Comprehensive geriatric assessments can provide critical insights that improve patient outcomes during chemotherapy.

## 1. Introduction

The introduction of gastrointestinal malignancies is a significant cause of morbidity and mortality worldwide [1]. In recent years, there has been increasing interest in assessing the body composition of patients with this type of cancer in order to perform nutritional evaluations and determine a prognosis. Sarcopenia is a condition characterized by the progressive and generalized loss of skeletal muscle mass, strength, and function during aging [2]. Numerous studies have demonstrated the adverse effects of sarcopenia on cancer outcomes; these include an increased risk of postoperative complications, a prolonged hospital stay, poor quality of life, intolerance to anticancer therapy, and decreased overall survival [3,4,5,6]. Accurate assessment and timely intervention may prevent or mitigate chemotherapy-related muscle toxicity, improving treatment outcomes and patients’ quality of life [5,7,8]. Sarcopenia is often multifactorial, especially in cancer patients. It can be affected by the cancer itself (through mechanisms such as cancer cachexia), nutritional deficiencies, decreased physical activity, and the aging process. The toxic effects of chemotherapy, such as nausea, vomiting, and fatigue, can lead to reduced food intake and decreased physical activity, which contributes to muscle wasting. Therefore, observing the same person before and after chemotherapy could aid in examining the relationship between chemotherapy and sarcopenia.

Some articles addressing the relationship between sarcopenia and chemotherapy define sarcopenia as low muscle mass, as measured via computed tomography, but do not evaluate muscle strength and performance. However, according to the new European Working Group on Sarcopenia of Older People (EWGSOP-2) 2018 criteria, it is not appropriate to diagnose sarcopenia by measuring muscle mass alone [2]. It has become essential to investigate the impact of other components of sarcopenia (the combination of low muscle mass plus low muscle strength or low physical performance) that determine the actual functionality.

The aim of this study was to examine whether sarcopenia developed in patients with gastrointestinal cancer who were receiving chemotherapy treatment and whether their condition changed compared to before chemotherapy. We also wanted to investigate whether chemotherapy affected the comprehensive geriatric assessment results of older participants.

## 2. Materials and Methods

### 2.1. Study Participants

**Figure 1:**
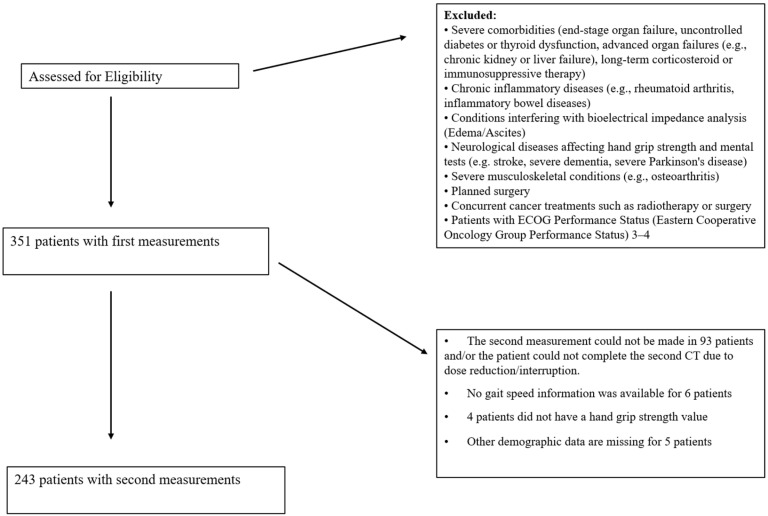
Patient selection flow chart.

This cross-sectional study included 351 patients diagnosed with gastrointestinal cancer at Ankara Numune Training and Research Hospital, Gazi University Hospital, and Ankara Atatürk Training and Research Hospital between October 2018 and December 2019. Measurements were taken before chemotherapy, and second measurements were obtained before the next dose. Due to the longitudinal nature of this study, which involves repeated measurements of the same participants before and after chemotherapy, a separate control group was not included. Instead, each participant served as their own control, allowing for the direct assessment of chemotherapy-related changes. This design eliminates inter-individual variability and ensures that the differences observed reflect within-subject changes over time. Out of the initial sample, 243 patients participated in the post-chemotherapy measurement. The study included patients with cancer at any stage who were not scheduled for surgery during the follow-up period. Details of the patient selection process are presented in Figure 1.

Table 1 and Table 2 present the demographic information collected for all participants. Demographic data (age, gender, cancer type, treatment protocol, stage, and BMI (kg/m^2^) (BMI was calculated as weight (kg) divided by the square of height (m^2^)), gait speed (s/4 m), muscle mass (kg), hand grip strength (kg), albumin (g/L), measurement days), body components with bioelectrical impedance analysis (BIA), gait speed, and hand grip strength were measured for all participants before and after chemotherapy. A comprehensive geriatric assessment was also performed in the older group (≥65 y). The data of patients undergoing chemotherapy were compared pre- and post-treatment. In this study, we conducted sarcopenia measurements before and after chemotherapy in patients diagnosed with gastrointestinal cancer. Due to ethical considerations and patient safety concerns, it was not feasible to establish a control group that did not receive chemotherapy. The nature of our patient population and treatment protocols made it impossible to withhold chemotherapy for a comparative control group. To address this limitation and ensure the robustness of our findings, we employed rigorous statistical analyses and a comprehensive approach to data collection. The absence of a control group is acknowledged as a limitation and is discussed in detail in Section 4. Nevertheless, our study provides valuable insights into the impact of chemotherapy on sarcopenia and highlights the importance of early detection and intervention strategies. The pre-chemotherapy measurements were taken within 7 days before the initiation of chemotherapy, ensuring a baseline assessment. Post-chemotherapy measurements were collected immediately before the next chemotherapy dose, typically 3–4 weeks after the initial dose. The study was conducted between October 2018 and December 2019, with consistent timelines for all participants.

### 2.2. Sarcopenia Measurement

Sarcopenia was diagnosed according to the EWGSOP-2 criteria [2].

#### 2.2.1. Bioelectrical Impedance Analysis (BIA)

**Figure 2:**
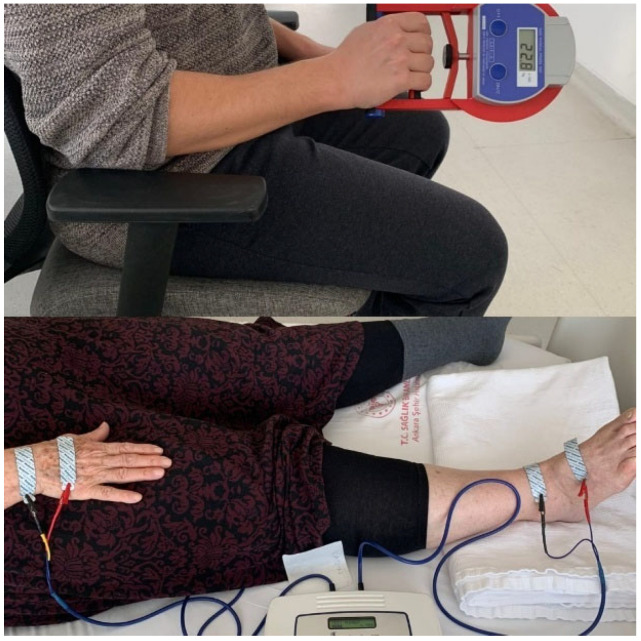
Assessment of handgrip strength and bioimpedance analysis.

The patients’ muscle mass was measured via BIA using a portable analyzer (Quadscan 4000, Bodystat, Douglas, Isle of Man, UK) in the supine position. Resistance was measured in ohms (Ω), and the device was adjusted according to the participant’s age, gender, height, and weight. Skeletal muscle mass (SMM) was calculated using the formula suggested by Janssen et al. [9]. Local cut-off values specific to the Turkish population were used: <9.2 kg/m^2^ for men and <7.4 kg/m^2^ for women [10]. Illustrations of the BIA measurement process are shown in Figure 2.

Permission to use photographs was obtained from participants. Top panel: Handgrip strength measurement using an electronic hand dynamometer (GRIP-D, Takei, Japan). Bottom panel: Bioelectrical impedance analysis (BIA) performed in the supine position using the Quadscan 4000 device for body composition assessment.

#### 2.2.2. Hand Grip Strength Test

Muscle strength was evaluated using a hand grip strength test performed with an electronic hand dynamometer (GRIP-D, Takei, Japan). The test was conducted three times using the dominant hand, and the average of the three measurements was recorded as the participant’s handgrip strength. Local cut-off values for grip strength were <22 kg for females and <32 kg for males [10]. An illustration of the hand grip strength test is shown in Figure 2.

#### 2.2.3. Gait Speed Test

**Figure 3:**
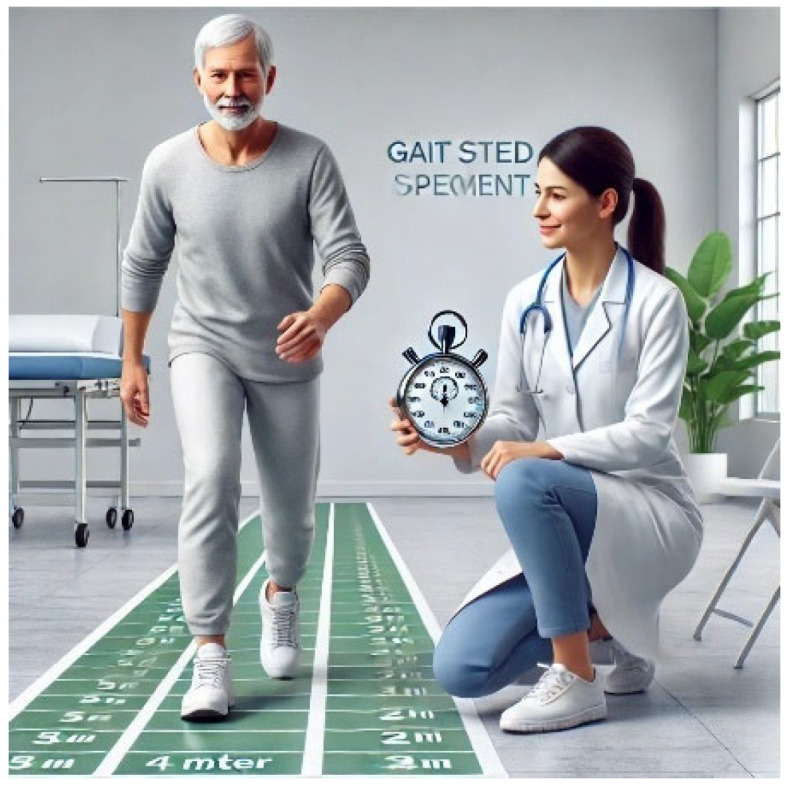
Gait speed illustration. Figure created using AI-generated tools provided by OpenAI’s DALL·E model.

Physical performance was assessed by measuring gait speed (m/s). Participants walked a 4 m course, and their walking time was measured using an electronic stopwatch. Gait speed was then calculated using the formula: 4 m/walking time (seconds). A gait speed of ≤0.8 m/s indicated reduced physical performance. The gait speed test setup is illustrated in Figure 3.

Patients with low muscle strength were categorized as having “possible sarcopenia”. When low muscle mass was also detected, sarcopenia was diagnosed. If low physical performance (as indicated by reduced gait speed) was present in addition to low muscle mass and strength, severe sarcopenia was diagnosed.

As recommended by the EWGSOP-2 guidelines, local cut-off values were utilized for sarcopenia diagnosis, incorporating thresholds specific to the Turkish population: grip strength <22 kg for females and <32 kg for males, based on regional reference data [10]. Skeletal muscle mass was evaluated with BIA. Those with low muscle strength were defined as possible sarcopenia. If the measurements indicated low muscle strength (low skeletal muscle mass), sarcopenia was diagnosed. If low physical performance was added to this, severe sarcopenia was diagnosed.

### 2.3. Comprehensive Geriatric Assessment

Comprehensive geriatric assessment included the Katz Activities of Daily Living Index (ADL), the Lawton Instrumental Activities of Daily Living Scale (IADL), the Mini-Mental Status Examination (MMSE), and the Mini-Nutritional Assessment Short Form (MNA-SF).

#### 2.3.1. Katz ADL Index

This index evaluates daily activities such as dressing, bathing, going to the toilet, getting out of bed, eating, and continence, with a maximum score of 6 points [11].

#### 2.3.2. Lawton IADL Scale

This scale assesses instrumental activities such as telephone use, shopping, food preparation, household chores, laundry, urban transportation, and proper use of medications, with a maximum score of 8 points [12].

#### 2.3.3. MMSE

Cognitive functions were evaluated using the MMSE, which has a maximum score of 30 points. Lower scores indicate cognitive impairment [13].

#### 2.3.4. MNA-SF

Nutritional status was assessed using the Mini-Nutritional Assessment Short Form.

### 2.4. Statistical Analysis

The sample size was calculated using the G*Power 3.1.9.7 program to ensure the study was adequately powered to detect significant differences. Assuming that the prevalence of sarcopenia would increase from 20% to 30% post-chemotherapy [14], with a power of 80% and an alpha of 0.05, a minimum of 87 participants were required. Our study included 243 participants, exceeding the minimum sample size required and ensuring sufficient power for statistical analysis.

The Statistical Package for the Social Sciences (SPSS) for Windows version 24.0 (IBM SPSS Inc., Chicago, IL, USA) was used for data analysis. The conformity of the variables to a normal distribution was examined using visual methods (histograms and probability graphs) and analytical tests, including the Kolmogorov–Smirnov and Shapiro–Wilk tests. Descriptive statistics were presented as mean ± standard deviation for normally distributed variables and as median, minimum–maximum, and interquartile range (IQR) for non-normally distributed variables. The frequency of categorical variables was expressed in percentages (%). The Wilcoxon signed-rank test was used to compare paired non-parametric data (e.g., pre- and post-chemotherapy measurements) due to its robustness against non-normal distributions (as shown in Table 2). The McNemar test was used to assess changes over time (as shown in Table 3) for dependent categorical variables. Pearson correlation analysis was used to evaluate the relationships between normally distributed numerical variables, while Spearman correlation was applied to non-normally distributed numerical variables. The Bonferroni correction was applied where appropriate to control for Type I errors due to multiple comparisons. The results were assessed within a 95% confidence interval, and a *p*-value of <0.05 was considered statistically significant.

### 2.5. Ethics Committee Approval

This study protocol was approved by the Ethics Committee of Ankara Numune Teaching Hospital (Approval No: E-770821-17.01.02). Before participation, all patients provided written informed consent.

## 3. Results

The median age of 243 patients was 57.84 (IQR: 26–79). The female gender ratio was 31.7% (n: 77). In addition, 29.2% (n: 71) of the participants were 65 and older. The demographic data of the participants are summarized in Table 1.

An initial evaluation of the patients was performed before treatment. Measurements were taken before and after chemotherapy. An analysis compared the patients’ pre-chemotherapy (preCT) and post-chemotherapy (postCT) measurements, which had a time gap of 62.06 ± 25.61 days. The postCT values for BMI and muscle mass were significantly lower, indicating a decrease in these parameters. Additionally, there was a significant increase in the rate of sarcopenia after chemotherapy. Both the muscle mass and albumin levels of older patients decreased significantly postCT, while the prevalence of sarcopenia increased considerably. The Lawton–Brody score also decreased significantly; however, no notable differences were found in the geriatric assessment tests (Table 2).

A significant difference in sarcopenia groups before (preCT) and after chemotherapy (postCT) was observed. Of the 105 patients, 43.2% were classified as usual before chemotherapy. In contrast, 7.4% had probable sarcopenia, 16.9% had confirmed sarcopenia, and 4.1% had severe sarcopenia after chemotherapy, resulting in a total of 28.4% being diagnosed with some form of sarcopenia post-treatment. This finding was statistically significant (Z:88.93, *p* < 0.001). Detailed data can be found in Table 3.

In additional analyses of 71 older people who participated in the study, it was determined that of the 30 patients who did not have sarcopenia preCT, 23 had sarcopenia postCT to varying degrees. The difference between the two measurements was statistically significant (McNemar test, Z: 15.67, *p*: 0.016). No significant difference was found between these two groups when comparing comprehensive geriatric tests pre- and post-CT (for Wilcoxon test, for Katz Z: 0.102 *p*: 0.989, Lawton–Brody: −0.270 *p*: 0.787, for MNA-SF Z:-.040 *p*: 0.978, for MMSE Z: −247 *p*: 0.805)

**Figure 4:**
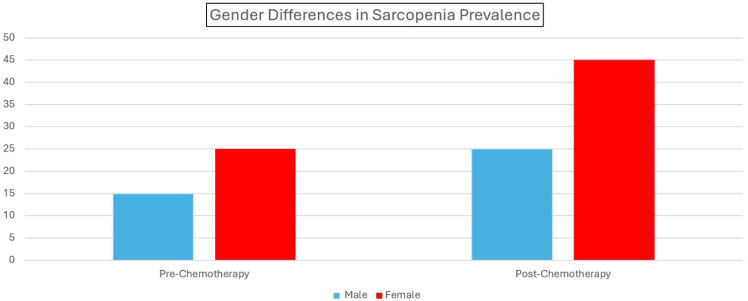
Gender differences in sarcopenia prevalence before and after chemotherapy. Comparison of sarcopenia prevalence between male and female patients before and after chemotherapy, highlighting the increase in sarcopenia rates across both genders.

**Figure 5:**
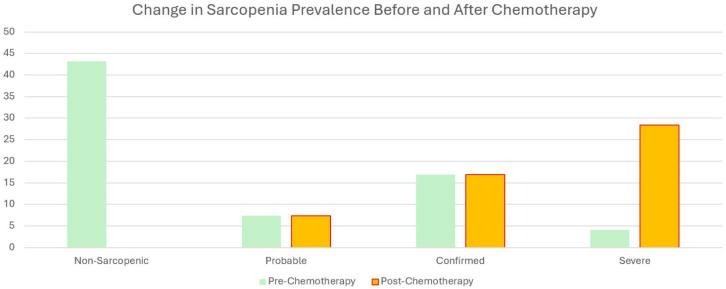
Changes in sarcopenia categories before and after chemotherapy. Comparison of sarcopenia prevalence across different categories (non-sarcopenic, probable, confirmed, and severe) before and after chemotherapy, highlighting the increase in confirmed and severe sarcopenia cases.

The main results of this study are summarized more clearly in Table 4, Figure 4 and Figure 5 below.

There was a significant increase in sarcopenia prevalence and a notable decrease in handgrip strength and gait speed after chemotherapy, highlighting the negative impact of chemotherapy on muscle function and physical performance.

## 4. Discussion

Our study highlights significant findings regarding the impact of chemotherapy on sarcopenia and associated parameters in patients with gastrointestinal cancer. Among the 243 participants, the post-chemotherapy evaluations revealed significant reductions in BMI and muscle mass, alongside a substantial increase in the prevalence of sarcopenia. The proportion of patients with probable, confirmed, or severe sarcopenia rose from 7.4%, 16.9%, and 4.1%, respectively, to a combined 28.4%. These effects were particularly pronounced in the older subgroup, where 23 of the 30 previously non-sarcopenic patients developed sarcopenia post-treatment. Despite these changes, no significant differences in the comprehensive geriatric assessment scores were observed, except for a decline in the Lawton–Brody scores, indicating reduced physical independence. These findings underline the clinical significance of sarcopenia as a critical outcome of chemotherapy.

### 4.1. Possible Mechanisms Underlying Sarcopenia Etiology

The mechanisms underlying sarcopenia in chemotherapy patients are complex and include systemic inflammation, metabolic derangement, and treatment-related side effects. Elevated levels of proinflammatory cytokines, such as IL-6 and TNF-α, disrupt anabolic signaling pathways, such as the IGF-1 axis, promoting muscle catabolism. These inflammatory responses, combined with chemotherapy-induced anorexia, fatigue, and hormonal imbalances, impair synthesis and exacerbate protein degradation. Older adults face greater risks than younger adults due to age-related declines in mitochondrial function and regenerative capacity. Chemotherapy-induced mitochondrial dysfunction reduces the production of cellular energy, compounding muscle weakness and fatigue. These processes demonstrate the multifactorial interactions between aging, chemotherapy, and sarcopenia, highlighting the need for comprehensive management strategies that address metabolic and inflammatory components [15,16,17].

### 4.2. Strengths and Main Findings of the Study

One of the strengths of this study is the use of the revised EWGSOP-2 criteria, which combines assessments of muscle mass, strength, and functionality for the diagnosis of sarcopenia. This multidimensional approach, with population-specific cut-off values, allowed for a more accurate assessment of sarcopenia in the Turkish population. Notably, many patients initially classified as normal before chemotherapy transitioned to a sarcopenic state after treatment, and some progressed to more severe stages. These findings are consistent with previous studies [18,19,20,21] and reinforce the role of chemotherapy in accelerating the progression of sarcopenia through catabolic and metabolic deterioration [5,6,22,23]. While decreases in handgrip strength and walking speed were observed, these changes were not statistically significant. This may be due to the relatively short interval between measurements, which was an average of 60 days; when comparing measurements, this interval may not have adequately captured functional declines. Other factors, such as unequal gender representation, a reliance on numerical thresholds rather than specific cut-offs, and small subgroup sizes, may have also contributed. It will be important to address these limitations in future studies using standardized measurements, longer follow-up periods, and larger cohorts.

### 4.3. Frailty in Older Patients

Our findings revealed that older patients were affected more than younger patients, with 23 out of 30 patients becoming sarcopenic after chemotherapy. This subgroup exhibited a significant decrease in their muscle mass and albumin levels. Our study once again emphasized that comprehensive geriatric and functional assessments should be performed in older people before chemotherapy, frailty screening tools should be used to detect wrinkling, and chemotherapy decisions should not be based solely on patients’ ECOG status. Age-related sarcopenia, combined with chemotherapy-induced fatigue and reduced physical activity, highlights the urgent need for personalized interventions. Resistance training, nutritional supplementation, and the close monitoring of functional indicators such as handgrip strength and gait speed are critical to reduce these risks [8,24,25].

### 4.4. Functional Measures and Their Implications

Handgrip strength and gait speed are established predictors of mortality and functional decline in cancer patients. Our findings reinforce their importance as key markers of sarcopenia and functional impairment. However, the lack of standard thresholds for cancer patients limits their applicability. Establishing robust, population-specific cut-off points for these parameters in the oncology setting should be a priority for future research [21,24,26,27].

### 4.5. Comprehensive Geriatric Assessments

This study also highlights the usefulness of comprehensive geriatric assessments (CGAs) in assessing the impact of chemotherapy on older adults. Significant decreases in Lawton–Brody scores after chemotherapy suggest a decline in the instrumental activities of daily living, even when other functional measures such as handgrip strength and gait speed appear unchanged. These findings highlight the value of CGAs in identifying subtle functional impairments in older patients undergoing chemotherapy and guiding interventions that maintain patients’ independence and quality of life. Sarcopenia is a well-recognized predictor of adverse outcomes, including mortality, in cancer patients. Its multifactorial and progressive nature, particularly in older adults, necessitates a proactive approach that encompasses early detection and personalized care strategies [28]. Incorporating resistance training and tailored nutritional plans into standard care protocols for cancer patients may significantly improve outcomes [29,30].

### 4.6. Strengths of the Study

In our study, we used the EWGSOP-2 criteria, which include muscle mass, strength, and functionality, to diagnose sarcopenia. This provided a more comprehensive and accurate assessment using a multidisciplinary approach. Applying cut-off values specific to the Turkish population increased the study’s validity in the regional context and allowed for more sensitive results.

Our study was designed prospectively, meaning that measurements were performed on the same individuals before and after chemotherapy. This enabled the direct observation of individual changes and reduced the margin of error in comparisons. Our study included biochemical data (e.g., albumin levels), muscle mass, muscle strength (handgrip strength), and functional parameters (gait speed). This provided a rich dataset for a multidimensional analysis of sarcopenia. We particularly focused on older patients and emphasized the vulnerability of this group to the development of sarcopenia after chemotherapy. We highlighted the need for special intervention strategies for older individuals. The evaluation of older patients before and after chemotherapy using the Comprehensive Geriatric Assessment (CGA) enabled us to not only analyze sarcopenia, but also the loss of independence in daily life activities. The findings of this study directly contribute to clinical practice by emphasizing the importance of hand grip strength and walking speed in diagnosing and managing sarcopenia during chemotherapy. Our study was designed with a multicenter approach that included data and methods from oncology, geriatrics, and nutrition, thus presenting more comprehensive results.

### 4.7. Limitations of the Study

However, the limitations of our study should be considered when interpreting the results. For example, the average follow-up period was only 60 days due to the heterogeneity of cancer and treatment types. This period may not be sufficient to fully capture the long-term effects of chemotherapy on sarcopenia and functional outcomes. Longer follow-up periods may provide a more comprehensive understanding of these effects. Specific follow-ups for a single cancer or treatment type may be more informative.

Another limitation is the paucity of biochemical data. While albumin levels were measured and included as part of the nutritional assessment, more advanced biochemical data, such as specific vitamin levels and other metabolic markers, were unfortunately not collected at the beginning of the study. We could not address this limitation due to the national health data system requiring personal SMS consent to access historical records.

In addition, although the overall sample size was sufficient, certain subgroups, such as older adults or specific genders, were underrepresented. This may limit the generalizability of the findings specific to these populations.

In addition, this study did not include a separate control group due to ethical and practical constraints. Instead, each participant served as their own control via pre- and post-chemotherapy measurements. This approach minimizes interindividual variability but may limit the broader generalizability of the findings. Mini Nutritional Assessment (MNA) testing and functional performance ECOG scores and BMI measurements were performed to assess the confounding factors. However, the lack of other advanced nutritional and performance measures may limit the depth of insights into the multifactorial nature of sarcopenia. While important, these limitations do not undermine the study’s primary findings. Instead, they highlight areas that could be addressed in future research to broaden our understanding of the relationship between chemotherapy and sarcopenia.

## 5. Conclusions

This study highlights the significant association between chemotherapy and sarcopenia in patients with gastrointestinal malignancies, emphasizing its impact on patient outcomes. The findings underscore the necessity of early detection and targeted interventions that address sarcopenia in cancer patients undergoing chemotherapy. Evaluating patients’ nutritional status, physical activity levels, and baseline sarcopenia indicators during diagnosis is crucial for assessing risk and implementing preventative measures.

To build upon these findings, future research should focus on the following:

- Conducting well-controlled, double-blind, prospective studies to establish causal relationships between chemotherapy and sarcopenia.
- Developing and testing specific intervention strategies, such as resistance training programs, dietary supplementation, and pharmacological treatments, to mitigate sarcopenia during chemotherapy.
- Expanding research to include patients with other cancer types and stages to assess the generalizability of these findings.
- Investigating the role of biochemical markers, including CRP, IL-6, and myostatin, in predicting and monitoring the progression of sarcopenia.
- Implementing longitudinal studies with extended follow-up periods to evaluate the long-term functional and quality-of-life outcomes associated with sarcopenia in cancer patients.

Future studies should aim to understand sarcopenia’s relationship with oncology by addressing these gaps and contributing to the development of effective management strategies. This will ultimately improve cancer patients’ quality of life and treatment outcomes.

## Figures and Tables

**Table 1:** The demographic data of the participants.

Comorbidities	n (%)
Diabetes mellitus	39 (16.0)
Hypertension	58 (23.9)
Heart failure	2 (0.8)
Cerebrovascular accident	3 (1.2)
Chronic renal failure	1 (0.4)
Coronary artery disease	16 (6.6)
Smoking	108 (44.4)
**Cancer Types**	
Gastric cancer	102 (42.0)
Esophageal cancer	6 (2.5)
Colon cancer	110 (45.3)
Pancreatic cancer	3 (1.2)
Other gastrointestinal cancer	21 (9.0)
**Treatment Protocols**	
FOLFOX	105 (43.2)
DCF	70 (20.8)
XELOX	1 (0.4)
FOLFIRI	7 (2.9)
Folinic acid 5-fluorouracil	14 (5.8)
Cisplatin 5-FU-docetaksel	16 (6.6)
Others ^1^	30 (12.2)
**Stage**	
Operated early stage	82 (44.6)
Non-operated locally advanced	34 (18.5)
Metastatic	68 (37.0)

Abbreviations: FOLFOX: Folinic acid + Fluorouracil + Oxaliplatin); DCF: Docetaxel-cisplatin-5-fluorouracil; XELOX: capecitabine + oxaliplatin; FOLFIRI: leucovorin calcium (folinic acid), fluorouracil, and irinotecan hydrochloride; ^1^: 5FU + Cisplatin (n = 1), Carboplatin-Etoposide (n = 3), Capecitabine + Oxaliplatin (n = 5), 5FU + Irinotecan (n = 6), Gemcitabine + Cisplatin (n = 7), and 5FU + Oxaliplatin + Docetaxel (n = 8).

**Table 2:** Comparison of sarcopenia components and comprehensive geriatric assessment for older patients preCT and postCT.

*	PreCT Median (IQR)	PostCT Median (IQR)	Z	*p*
**All Patients n: 243 (100%)**				
BMI (kg/m^2^)	25.9 (16.7–45.3)	25.6 (15.1–45.3)	−2.86 ^a^	**0.004**
Gait speed (s/4 m)	4.32 (2.40–8.00)	4.29 (2.02–10.50)	−0.191 ^b^	0.849
Muscle mass (kg)	8.55 (5.55–28.35)	7.56 (4.23–25.8)	−9.437 ^a^	**<0.001**
Hand grip strength (kg)	26.84 (7.50–53.51)	25.6 (10.73–51.23)	−0.109 ^b^	0.914
Sarcopenia (n/%) **	7 (2.9)	130 (53.7)		**<0.001**
Albumin (g/L)	4.11 (2.1–4.9)	4.00 (2.8–4.3)	−0.429 ^a^	0.663
**≥65 years n: 71 (29.2%)**				
BMI (kg/m^2^)	26.60 (18.80–45.30)	25.60 (17.6–45.30)	−0.378 ^a^	0.705
Gait speed (s/4 m)	4.99 (2.72–8.00)	8.34 (4.97–9.76)	−0.493	0.622
Muscle mass (kg)	8.93 (6.15–28.35)	8.34 (4.87–25.88)	−3.98 ^a^	**<0.001**
Hand grip strength (kg)	25.08 (9.93–45.27)	24.68 (10.73–43.13)	−0.0786 ^a^	0.432
Sarcopenia (n/%) **	4 (5.6)	37 (52.1)		**0.018**
Albumin (g/L)	4.11 (2.8–4.9)	4.03 (2.8–4.7)	−2.06 ^a^	**0.039**
Katz ADL	6 (4–6)	6 (2–5)	−0.405 ^b^	0.685
Lawton–Brody IADL	7 (1–8)	6 (2–8)	−2.03 ^b^	**0.042**
MNA-SF	10 (6–14)	9 (5–14)	−1.60 ^b^	0.978
MMSE	26 (18–30)	25 (16–30)	−1.35 ^a^	0.176

* Wilcoxon test applied; ** Chi-square test applied; ^a^: based on positive ranks; ^b^: based on negative ranks; Abbreviations: IQR: interquartile range; BMI: body mass index; ADL: activities of daily living; IADL: instrumental activities of daily living scale MNA-SF: mini-nutritional assessment-short form; MMSE: mini-mental status examination.

**Table 3:** Comparison of sarcopenia components and comprehensive geriatric assessment for older patients preCT and postCT.

		Post-Chemotherapy Sarcopenia Groups *				
		Normal n (%)	Probable Sarcopenia n (%)	Confirmed Sarcopenia n (%)	Severe Sarcopenia n (%)	Total n (%)
Pre-chemotherapy Sarcopenia groups	Normal n (%)	36 (14.8)	18 (7.4)	41 (16.9)	10 (4.1)	105 (43.2)
	Probable sarcopenia n (%)	3 (1.2)	10 (4.1)	6 (2.5)	6 (2.5)	25 (10.3)
	Confirmed sarcopenia n (%)	0 (0)	3 (1.2)	3 (1.2)	14 (5.8)	20 (8.2)
	Severe sarcopenia n (%)	4 (1.6)	19 (7.8)	58 (23.9)	12 (4.9)	93 (38.3)
	Total n (%)	43 (17.7)	50 (20.6)	108 (44.4)	42 (17.3)	243 (100)

* Categorical variables were evaluated using the McNemar test.

**Table 4:** Changes in Sarcopenia-Related Parameters Before and After Chemotherapy.

Parameter	Pre-Chemotherapy	Post-Chemotherapy	*p*-Value
Sarcopenia (%)	20	35	<0.001
Handgrip strength (kg)	28.5 ± 5.1	22.4 ± 6.3	0.002
Gait speed (m/s)	0.9 ± 0.1	0.6 ± 0.2	0.001

## Data Availability

For reasons of privacy and ethics, access to the data are restricted.
